# Inbreeding and runs of homozygosity before and after genomic selection in North American Holstein cattle

**DOI:** 10.1186/s12864-018-4453-z

**Published:** 2018-01-27

**Authors:** Mehrnush Forutan, Saeid Ansari Mahyari, Christine Baes, Nina Melzer, Flavio Schramm Schenkel, Mehdi Sargolzaei

**Affiliations:** 1 Department of Animal Science, College of Agriculture, Isfahan University of Technology, Khomeyni Shahr, Iran; 2 Centre for Genetic Improvement of Livestock, Department of Animal Biosciences, University of Guelph, Guelph, Canada; 3 Institute of Genetics and Biometry, Leibniz Institute for Farm Animal Biology, Dummerstorf, Germany; 4 Semex Alliance, Guelph, Canada; 5 HiggsGene Solutions Inc., Guelph, Canada

**Keywords:** Runs of homozygosity, Genomic selection, Inbreeding, BLUP selection

## Abstract

**Background:**

While autozygosity as a consequence of selection is well understood, there is limited information on the ability of different methods to measure true inbreeding. In the present study, a gene dropping simulation was performed and inbreeding estimates based on runs of homozygosity (ROH), pedigree, and the genomic relationship matrix were compared to true inbreeding. Inbreeding based on ROH was estimated using SNP1101, PLINK, and BCFtools software with different threshold parameters. The effects of different selection methods on ROH patterns were also compared. Furthermore, inbreeding coefficients were estimated in a sample of genotyped North American Holstein animals born from 1990 to 2016 using 50 k chip data and ROH patterns were assessed before and after genomic selection.

**Results:**

Using ROH with a minimum window size of 20 to 50 using SNP1101 provided the closest estimates to true inbreeding in simulation study. Pedigree inbreeding tended to underestimate true inbreeding, and results for genomic inbreeding varied depending on assumptions about base allele frequencies. Using an ROH approach also made it possible to assess the effect of population structure and selection on distribution of runs of autozygosity across the genome. In the simulation, the longest individual ROH and the largest average length of ROH were observed when selection was based on best linear unbiased prediction (BLUP), whereas genomic selection showed the largest number of small ROH compared to BLUP estimated breeding values (BLUP-EBV). In North American Holsteins, the average number of ROH segments of 1 Mb or more per individual increased from 57 in 1990 to 82 in 2016. The rate of increase in the last 5 years was almost double that of previous 5 year periods. Genomic selection results in less autozygosity per generation, but more per year given the reduced generation interval.

**Conclusions:**

This study shows that existing software based on the measurement of ROH can accurately identify autozygosity across the genome, provided appropriate threshold parameters are used. Our results show how different selection strategies affect the distribution of ROH, and how the distribution of ROH has changed in the North American dairy cattle population over the last 25 years.

**Electronic supplementary material:**

The online version of this article (10.1186/s12864-018-4453-z) contains supplementary material, which is available to authorized users.

## Background

Genomic selection, in which genetic markers across the whole genome are used to estimate breeding values of individuals, is routinely applied in dairy cattle breeding programs. In dairy cattle, genomic selection has resulted in a substantial increase in the rate of genetic gain compared to traditional selection [1]. This has been achieved mainly by reducing the generation interval, which became possible because of the higher reliabilities of genomic breeding values (GEBV) estimated early in life compared to parent averages. Genomic selection is expected to reduce the rate of inbreeding per generation by capturing Mendelian sampling variation more accurately than pedigree-based measures through reducing co-selection of relatives [2]. However, it can lead to a higher increase in the rate of inbreeding per years given the shorter generation interval, as young animals with high GEBV are selected to be parents [3]. Such higher increase could result in lower genetic variation, lower response to selection, and a higher risk of homozygosity for deleterious/lethal alleles [4]. The control of inbreeding rates and the maintenance of sufficient genetic diversity are important for the sustainability of selected dairy cattle populations.

Traditionally, inbreeding coefficients were estimated based on pedigree-based relationships (A) (e.g., [5]). For each individual, the inbreeding coefficient reflects the expected proportion of the genome that is autozygous. Pedigree-based (i.e. traditional) inbreeding is based on Mendelian sampling probabilities, so that the inbreeding coefficients of full-sibs are always identical. Using pedigree information for calculating the level of inbreeding usually underestimates the true inbreeding coefficient [6], due to incomplete pedigree information, especially for distant generations.

With the advent of high throughput genotyping technologies, interest has grown in using genomic information to estimate more precisely inbreeding coefficients [7]. Using single nucleotide polymorphism (SNP) markers, the realized proportion of the genome that is identical by state (IBS) or by descent (IBD) can be estimated for each individual. However, identifying the proportion of the genome which is identical by descent due to common ancestors beginning at a fixed time point requires making assumptions about allele frequencies at that time point [7, 8]. Several studies have shown that characterizing inbreeding based on long stretches of consecutive homozygous genotypes (runs of homozygosity; ROH) provides a better measure of individual autozygosity than estimating over-all inbreeding based on pedigree information [9–11]. ROH provide a better estimate of autozygosity at the genomic level and make it possible to identify specific IBD regions [12]. ROH regions can also be used to improve mating decisions and minimize unfavorable effects of inbreeding. Several studies have investigated the relationship between ROH and deleterious variants in livestock [13]. Zhang et al. [14] showed a higher frequency of deleterious variants compared to non-deleterious variants within ROH segments in dairy cattle. Those authors also showed that short (less than 100 kb) and medium (0.1 to 3 Mb) ROH regions have more deleterious variants than long (greater than 3 Mb) ROH regions.

Population structure and selection can be assessed based on ROH distribution and location. Long homozygous regions throughout the genome result from matings of close relatives, reduction in population size, and selection [15]. Overall, the similarity between individuals across the whole genome has increased in livestock as a consequence of selection [16, 17]. Kim et al. [17] reported that the distribution of ROH across the genome is more variable for selected than for unselected animals. The increase in autozygosity as a consequence of selection is well-understood. However, there is limited information on the effect of genomic selection on the distribution of ROH across the genome, and on the effect of genomic selection on the rate of inbreeding. Therefore, the objectives of this study were: 1) to determine the optimal method for estimating true inbreeding based on simulation, 2) to assess the effect of various methods of selection on true inbreeding and 3) to characterize the distribution of ROH across the genome of North American Holstein cattle before and after implementation of genomic selection.

## Results and discussion

### Simulation

#### Inbreeding analysis

##### Measures of inbreeding

True inbreeding (F_TRUE_) and inbreeding values based on pedigree (F_PED_), genomic relationship (F_GRM_) and ROH (F_ROH_) were calculated for all animals in simulated populations under four different selection criteria (random, phenotype, BLUP-EBV, and GEBV). The average inbreeding coefficients estimated using different approaches are presented in Tables 1 and 2. For simplicity, in both tables, only the F_ROH_ based on SNP1101 using a minimum window size of 20 SNP is presented, which resulted in the closest estimates to true simulated inbreeding.

**Table 1:** Different measures of inbreeding^a^: True inbreeding (F_TRUE_); inbreeding derived from a pedigree (F_PED_) and inbreeding estimated from the genomic relationship matrix using known base allele frequencies (F_GRM_Base_) or an allele frequency of 0.5 (F_GRM_Fixed_); and inbreeding estimated based on runs of homozygosity (F_ROH_) in simulated populations with equal base allele frequencies (*p* = 0.5)

	Phenotype selection			Random selection			GEBV selection^c^			BLUP-EBV selection^d^		
Generation	10	30	60	10	30	60	10	30	60	10	30	60
F_TRUE_	0.024	0.099	0.209	0.014	0.046	0.092	0.035	0.173	0.411	0.058	0.252	0.554
F_PED_	0.022	0.074	0.137	0.014	0.046	0.092	0.029	0.093	0.181	0.054	0.201	0.446
F_ROH_^b^	0.024	0.099	0.209	0.014	0.046	0.092	0.035	0.173	0.411	0.058	0.253	0.555
F_GRM_Fixed_	0.024	0.099	0.209	0.014	0.046	0.092	0.035	0.173	0.411	0.058	0.252	0.554
F_GRM_Base_	0.023	0.098	0.208	0.014	0.045	0.091	0.034	0.172	0.41	0.057	0.251	0.553

^a^Standard error for all estimates ranged from 0.001 to 0.0008

^b^F_ROH_based on SNP1101 (minimum homozygosity 20 SNP, genotype error rate 0.001)

^c^Selection based on genomic estimated breeding value

^d^Selection based on best linear unbiased prediction estimated breeding value

**Table 2:** Estimates^a^ of true inbreeding (F_TRUE_), inbreeding derived from runs of homozygosity (F_ROH_), inbreeding estimated from the genomic relationship matrix using known base allele frequencies (F_GRM_Base_) or an allele frequency of 0.5 (F_GRM_Fixed_) in simulated populations with uniform base allele frequencies (ranging from 0 to 0.5)

	Phenotype selection			Random selection			GEBV selection^c^			BLUP-EBV selection^d^		
Generation	10	30	60	10	30	60	10	30	60	10	30	60
F_TRUE_	0.024	0.099	0.209	0.014	0.046	0.092	0.035	0.173	0.411	0.058	0.252	0.554
F_ROH_^b^	0.026	0.102	0.214	0.017	0.049	0.095	0.038	0.177	0.418	0.060	0.256	0.561
F_GRM_Fixed_	0.293	0.344	0.422	0.030	0.320	0.350	0.291	0.387	0.562	0.293	0.433	0.661
F_GRM_Base_	0.030	0.111	0.224	0.018	0.053	0.101	0.045	0.192	0.435	0.074	0.283	0.593

^a^Standard error for all estimates ranged from 0.001 to 0.0008

^b^F_ROH_based on SNP1101 (minimum homozygosity 20 SNP, genotype error rate 0.001)

^c^Selection based on genomic estimated breeding value

^d^Selection based on best linear unbiased prediction estimated breeding value

##### Pedigree-based inbreeding coefficient

F_PED_ may not be an accurate measure of inbreeding in populations under selection. The F_PED_ estimates were lower than F_TRUE_ in selected populations. The pedigree-based inbreeding assumes neutral loci, i.e. that the two alleles at the same locus on two homologous chromosomes have an equal chance of being selected. In reality for some loci the two alleles may have different effects on a naturally or artificially selected trait, which leads to unequal selection probabilities between the two alleles [18]. As a result, selection on a trait controlled by a few quantitative trait locus (QTL) with large effects or on a complex trait controlled by a large number of QTL but with limited genome size will change the allele frequencies [19]. In agreement with our results, Liu et al. [19], using a simulation study, reported that pedigree-based inbreeding could not accurately reflect the rate of true inbreeding. Villanueva et al. [20] reported that pedigree-based inbreeding might be a good estimate of true inbreeding only under an infinitesimal model, because the discrepancy between pedigree and true inbreeding over all QTL decreases with the number of QTL.

The correlation between F_TRUE_ and F_PED_ decreased as the number of generations increased in selected populations. The correlations between F_PED_ and F_TRUE_ were 0.79, 0.61, and 0.50 in generation 10, 30 and 60 of GEBV selection, respectively. These values were 0.78, 0.60, and 0.49 when selection was based on BLUP-EBV and 0.82, 0.66, and 0.57 for phenotypic selection, respectively. This result was expected because directional selection can reduce genetic diversity surrounding the QTL as result of “hitch-hiking” [21]. Hitch-hiking acts as an important mechanism to reduce genetic diversity and increase the rate of inbreeding further by gradually decreasing frequency of linked neutral polymorphisms in the population.

The difference between F_PED_ and F_TRUE_ was the highest for long-term GEBV selection versus traditional selection (Table 1). Genomic selection causes stronger selection pressure for the QTL and leads to faster QTL fixation. Liu et al. [19] stated that the strength of selection of the QTL may be an essential factor for the level of hitch-hiking observed for each selection criteria. Therefore, F_PED_ does not accurately reflect the rate of true inbreeding in a population under genomic selection.

##### Genomic relationship-based inbreeding coefficient

Genomic inbreeding coefficients are dependent on assumptions about allele frequencies in the base population [7]. These frequencies are usually unknown, therefore their choice is a challenging problem. In the US, base allele frequencies of 0.5 are used for calculating genomic inbreeding values. Bjelland et al. [22] suggested using base frequencies of 0.5 in the base population. VanRaden et al. [23] indicated that using allele frequencies of 0.5 resulted in higher correlations between F_GRM_ and F_PED_.

In the present study, two base populations with initial allele frequencies either equal to 0.5 (scenario 1) or sampled from a uniform distribution (ranging from 0 to 0.5; scenario 2) for founder animals were simulated. For both scenarios, genomic inbreeding coefficients were computed with a formula that used true allele frequency obtained from the base population (F_GRM-Base_), and equal allele frequency (*P* = 0.5; F_GRM-Fixed_). Table 1 contains corresponding results for scenario 1 and Table 2 for scenario 2. As expected, when known base allele frequencies were used, estimates of genomic inbreeding were closer to true inbreeding in both scenarios. In scenario 1, the F_GRM-Base_ and F_GRM-Fixed_ estimates were close to true inbreeding. In contrast, in scenario 2 the F_GRM-Fixed_ was clearly overestimated, although the correlation between F_TRUE_ and F_GRM-Fixed_ was close to one (Table 2). Bjelland et al. [22] reported correlations of 0.81 between F_ROH_ and F_GRM_ when an allele frequency of 0.5 was used for calculating F_GRM,_ and a correlation of 0.55 if base allele frequency was estimated. This would suggest that using a simple allele frequency of 0.5 may be more beneficial than attempting to estimate them in the base population. In our study F_GRM-Fixed_ overestimated true inbreeding when base allele frequencies deviated from 0.5, it seems more appropriate to use F_ROH_ for an accurate estimation of true inbreeding.

##### ROH-based inbreeding coefficients

Estimates of ROH-based inbreeding were obtained using different software and different window sizes, SNP1101 (with window sizes of 5, 20, 35 and 50 SNPs), PLINK (with window sizes of 20, 35 and 50 SNPs) and BCFtools (does not use a window approach) (Table 3). Defining different minimum length of ROH is analogous to changing the depth of pedigree or base population in pedigree inbreeding. Shorter ROH display more ancient inbreeding, while longer ROH show more recent inbreeding [24]. When minimum window size was 5 SNPs (approximately 0.25 Mb) the inbreeding rates were overestimated in all generations (Table 3). The overestimation could be explained by observing many short segments that are homozygote by chance. The overestimation was smaller in older generations due to the fact that occurrence of small size ROH by chance depends on the number of recombination events since the base population, which is a function of the number of generations.

**Table 3:** Estimates^a^ of true inbreeding (F_TRUE_) and runs of homozygosity based inbreeding using PLINK (F_PLINK_), SNP1101 (F_SNP1101_), BCFtools (F_BCFtools_) in simulated populations with equal base allele frequencies (*p* = 0.5)

Selection method		Phenotype selection			Random selection			GEBV selection^d^			BLUP-EBV selection^e^		
Generation		10	30	60	10	30	60	10	30	60	10	30	60
F_TRUE_		0.024	0.099	0.209	0.014	0.046	0.092	0.035	0.173	0.411	0.058	0.252	0.554
F_BCFtools_		0.024	0.099	0.208	0.025	0.046	0.091	0.035	0.172	0.409	0.058	0.251	0.552
F_SNP1101_^b^	S1	0.062	0.134	0.241	0.052	0.083	0.128	0.072	0.206	0.437	0.094	0.282	0.574
	S2	0.024	0.099	0.209	0.014	0.046	0.092	0.035	0.173	0.411	0.058	0.253	0.555
	S3	0.024	0.098	0.204	0.014	0.046	0.089	0.035	0.172	0.404	0.058	0.251	0.550
	S4	0.024	0.097	0.198	0.014	0.045	0.087	0.035	0.169	0.395	0.057	0.248	0.542
F_PLINK_^c^	P1	0.024	0.094	0.194	0.024	0.044	0.085	0.034	0.165	0.387	0.057	0.242	0.532
	P2	0.024	0.094	0.191	0.025	0.044	0.084	0.034	0.164	0.382	0.056	0.241	0.528
	P3	0.024	0.092	0.186	0.025	0.043	0.081	0.034	0.162	0.375	0.056	0.238	0.522

^a^Standard error for all estimates ranged from 0.001 to 0.0008

^b^S1: min homozygosity 5 SNP; S2: min homozygosity 20 SNP; S3: min homozygosity 35 SNP; S4: min homozygosity 50 SNP, S1-S4: the genotype error rate was 0.001

^c^P1: homozyg-window-snp 20, homozyg-snp 20; P2: homozyg-window-snp 35, homozyg-snp 35; P3: homozyg-window-snp 50, homozyg-snp 50, P1-P3: homozyg-window-het 1

^d^Selection based on genomic estimated breeding value

^e^Selection based on best linear unbiased prediction estimated breeding value

Results showed that, with the SNP window set to 20–50 SNPs, F_ROH_ was more accurate than F_PED_ and F_GRM_, i.e. closer to the true inbreeding coefficients in all scenarios and under all selection criteria (Tables 1 and 3). Kim et al. [16] tested two minimum window sizes of 50 and 100 SNPs in 50 k chip data and concluded that using 50 SNP threshold was better for autozygosity detection and defining ROH derived from older common ancestors. Liu et al. [19] used sliding windows of 10, 25 or 50 SNPs and reported that, within this range, window length did not have a significant effect on the rate of ROH-based inbreeding. In agreement with current results, previous studies have shown that F_ROH_ is a better measure of individual autozygosity than pedigree-based inbreeding [9–11]. Keller et al. [6] indicated that ROH-based inbreeding is preferable to F_PED_ and other measures of genomic-based inbreeding, because it correlates strongly with homozygous mutation load.

ROH-based inbreeding coefficients obtained by SNP1101 and BCFtools were well correlated (close to 1) with F_TRUE_ for all scenarios. Estimates of inbreeding coefficients using PLINK with minimum window size 20–50 SNPs in the last 25 generations tended to be lower than true inbreeding in all populations (Table 3). The correlation between F_ROH_ calculated using PLINK and F_TRUE_ in generation 60 in all populations was about 0.98 (data not shown). Differences between SNP1101 and PLINK might be due to the different ways ROH length window were defined by each program. PLINK uses a fixed sliding window approach that moves along the chromosome one SNP at a time and searches along SNP data to detect homozygous stretches. In contrast, SNP1101 uses an overlapping sliding window approach to efficiently identify ROH segments from longest to shortest. The process starts with long window to capture ROH segments and, then, the window size is gradually reduced allowing for detection of shorter ROH segments. In contrast, BCFtools uses a hidden Markov model (HMM) to identify ROHs. It is designed to exploit all the information available from population genotype sequencing, which includes more information about allele frequencies and recombination rates [25]. Given that SNP1101 provide the most accurate estimates of inbreeding and requires considerably less computational time to detect the ROH segment compared to BCFtools, all further analyses for F_ROH_ were done using SNP1101 with a window size of 20 SNP.

##### The effect of different selection strategies on inbreeding

The highest average inbreeding was observed for selection based on BLUP-EBV, followed by GEBV, phenotypic and finally random selection (Table 1). In selected populations there are considerable differences between the genetic contributions of individuals with high genetic merit compared to animals with lower genetic merit. This increases the rate of inbreeding compared to an unselected population. BLUP-EBV is based on pedigree information and the phenotypic records of both selection candidates and their relatives. The use of family information in estimating breeding values increases the correlations between the EBVs of relatives. This also increases the chance of co-selection of relatives which, in turn, leads to an increase in the rate of inbreeding. Here we simulated a trait with a heritability of 0.3. When selection is based on BLUP-EBV, the rate of inbreeding decreases with increasing heritability [26]. In EBV estimation, there is more weight on pedigree information for low heritable traits compared to traits with high heritable traits. Therefore, for traits with lower heritability, the correlation between EBV of relatives is increased, thus resulting in a higher chance of co-selection of relatives, which ultimately leads to a higher rate of inbreeding per generation. EBV based on genomic information has a higher accuracy than traditional BLUP-EBVs because SNPs provide a more accurate estimate of relationship than pedigree. Therefore, individuals within a full sib family have different genetic merits, which results in decreasing co-selection of sibs, and consequently in lower inbreeding compared to BLUP-EBV. Selection on BLUP-EBV or GEBV result in smaller effective population size and, therefore, higher inbreeding compared to phenotype selection, which only uses the phenotypic record of the individual animal, or random selection.

##### Distribution of runs of Homozygosity

**Fig. 1:**
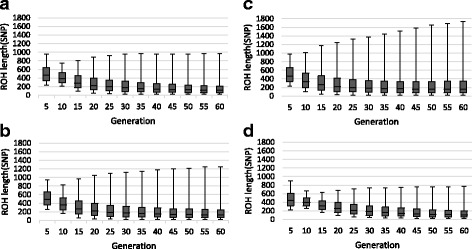
Box plots of the detected length of runs of homozygosity (ROH) over the generations using equal base allele frequencies (*p* = 0.5) for the different selection strategies: (**a**) Phenotype selection; (**b**) Genomic selection; (**c**) BLUP selection; (**d**) Random selection. For the analyses the SNP1101 (minimum window size = 20SNP, genotype error = 0.001) was used

Table 4 shows the ROH statistics at window size 20 SNP in the simulated populations. In the present study, all animals in the base generation were unrelated and, therefore, there were only a few short ROH segments, which appeared by chance in the generation zero. Mating of close relatives usually results in creation of long ROH segments, while the mating of animals with more distant relationship may result in shorter ROH segments. Matings resulting in ROH in initial generations were mostly between close relatives and, therefore, longer ROH segments were observed initially. These segments were broken down by meiotic recombination over generations and, therefore, the minimum and average length of ROH decreased over generations (Table 4). The average number of ROH and overall autozygosity per animal increased over generations in all populations. Comparisons of ROH distribution between selected or unselected populations for increasing favorable QTL revealed noticeable differences with respect to overall ROH frequency and length (Fig. 1). As expected, selection has increased overall autozygosity across the genome. In addition, ROH length was more variable in selected animals in comparison to a more even ROH length for unselected animals. Zhang et al. [14] reported that distribution of ROH over the genome is not random. They suggested that it is affected by selection. Current results confirm that selection plays an important role in determining and shaping the distribution of ROH. Based on current results, ROH analysis is a viable method for assessing the effect of selection on genome autozygosity.

**Table 4:** Summary statistics of runs of homozygosity (ROH) calculated in simulated populations with base allele frequencies of 0.5 over 20 replicates, using SNP1101 software (minimum window size = 20SNP, genotype error = 0.001)

Generation	Number of ROHs ± standard error	Minimum ROH length(SNP) ± standard error	Maximum ROH length(SNP) ± standard error	Average ROH length(SNP) ± standard error
	Random selection			
5	3.9 ± 0.05^a^	217.3 ± 6.21^a^	898.2 ± 15.00^a^	488.9 ± 8.00^a^
20	5.2 ± 0.05^a^	94.2 ± 1.19^d^	676.7 ± 4.95^a^	307.9 ± 1.25^b^
40	15.8 ± 0.12^a^	30.5 ± 0.21^d^	746.0 ± 5.13^a^	207.4 ± 0.89^a^
60	30.4 ± 0.24^a^	23.6 ± 0.11^d^	769.7 ± 4.50^a^	163.2 ± 0.41^a^
	Phenotype selection			
5	4.0 ± 0.07^a^	231.3 ± 7.93^a^	958.6 ± 14.48^b^	520.0 ± 8.98^b^
20	10.1 ± 0.21^b^	51.1 ± 0.92^c^	885.1 ± 9.97^b^	306.3 ± 1.65^ab^
40	34.8 ± 0.58^b^	24.4 ± 0.13^c^	959.8 ± 9.50^b^	212.7 ± 1.23^b^
60	66.0 ± 0.77^b^	21.6 ± 0.11^c^	971.8 ± 10.26^b^	172.1 ± 1.06^b^
	BLUP-EBV selection^1^			
5	4.2 ± 0.06^b^	230.9 ± 4.58^a^	973.71 ± 9.13^b^	527.2 ± 4.63^b^
20	25.4 ± 0.58^d^	30.8 ± 0.24^a^	1246.8 ± 18.90^d^	317.1 ± 3.30^c^
40	74.0 ± 0.82^d^	22.1 ± 0.09^a^	1511.7 ± 27.07^d^	264.4 ± 3.38^d^
60	110.6 ± 0.89^c^	21.2 ± 0.10^b^	1729.6 ± 18.29^d^	273.5 ± 3.03^d^
	GEBV selection^2^			
5	3.8 ± 0.07^a^	254.1 ± 5.07^b^	947.5 ± 8.92^b^	534.4 ± 3.43^b^
20	17.0 ± 0.42^c^	36.5 ± 0.46^b^	1050.2 ± 12.71^c^	303.3 ± 2.57^a^
40	62.9 ± 0.67^c^	22.4 ± 0.11^b^	1179.9 ± 14.49^c^	220.8 ± 1.91^c^
60	113.4 ± 0.90^d^	21.0 ± 0.00^a^	1242.9 ± 10.84^c^	197.3 ± 1.76^c^

^1^Selection based on best linear unbiased prediction estimated breeding value

^2^Selection based on genomic estimated breeding value

Different letters indicate statistical significance within the same equivalent generation and each column (*P* < 0.05)

**Fig. 2:**
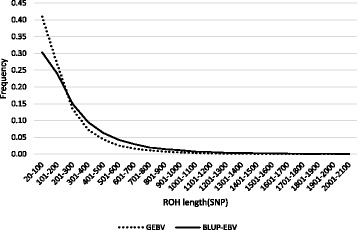
Frequency of runs of homozygosity (ROH) length in generation 30 in BLUP estimated breeding value (BLUP-EBV) and generation 60 in genomic breeding value (GEBV) based selection, using SNP1101 software (minimum window size = 20SNP, genotype error = 0.001)

As expected, the highest average ROH length and the longest ROH segments were observed when BLUP-EBV was used to select animals as parents of the next generation (Fig. 1). Considering, however, that the generation interval for genomic selection in dairy cattle is at least half of that for traditional selection, one can compare ROH distributions at generations 60 for GEBV and 30 for BLUP-EBV to assess the effect over a similar time scale. As can be seen in Fig. 2, the frequency of long ROH (greater than 300 SNP or approximately 15 Mb) was higher in BLUP-EBV selection compared to that in GEBV selection. On the other hand, the frequency of small and intermediate ROH (lower than 300 SNP) was higher in GEBV selection compared to BLUP-EBV. Estimates of breeding values based on genomic information are more accurate than BLUP-EBV for young animals because genomic relationships capture more Mendelian sampling variation compared to that captured by pedigree-based relationships. Therefore, selection based on GEBV compared to BLUP-EBV results in less related selection candidates, leading to a greater number of intermediate and small ROH, and a lower number of long ROH. It should be noted that the data simulation was carried out for a general population mainly aiming to generate true inbreeding for comparing different ROH estimation methods. Therefore, the present simulation did not aim at mimicking a real dairy cattle breeding program and care should be taken for not directly comparing the simulation results to real dairy cattle data.

#### Real data

##### Inbreeding coefficients

Three different estimates of inbreeding (F_PED_, F_GRM_ and F_ROH_) were calculated for each animal in a sample of genotyped North American Holstein animals born between 1990 and 2016. Given that SNP1101 provided most accurate estimates of inbreeding compared to PLINK and it took considerably less computational time to identify ROH segments compared to BCFtools, F_ROH_ was estimated based on a sliding window approach implemented in SNP1101 (minimum window size 20; genotype error 0.001).

Using real data, low density (≥7 k) genotypes of animals were imputed to medium density (i.e. 50 k). Pedigree information was included to achieve high imputation accuracy, and a large reference population of 50,000 animals with 50 k genotypes was used. Imputation is expected to have minimal effects on the results, as accuracy for such a scenario is expected to be higher than 99% on average [27].

The rate of pedigree-based inbreeding was lower than genomic-based inbreeding. The average inbreeding coefficients using genomic information was about 0.30 and 0.15 for F_GRM_ and F_ROH_, respectively, for animals born in 2016. The corresponding value for pedigree-based inbreeding was about 0.087 (Fig. 4). Several studies in cattle populations have also reported higher estimates of inbreeding coefficients when genomic information was used compared to pedigree information [10, 12]. Liu et al. [19] reported that the rate of inbreeding measured by pedigree does not accurately reflect the rate of true inbreeding. This can be explained by considering that F_PED_ is an expectation of the genome that is IBD, but there is much variation around this expectation because of the stochastic nature of recombination [6]. In addition, in calculation of the pedigree-based inbreeding coefficients, it is assumed that founder animals are unrelated. This assumption may lead to under-estimation of pedigree-based inbreeding if the recorded pedigree is not deep enough or is incomplete. Also as stated before, F_PED_ assumes that the loci are neutral and, therefore, it does not consider potential bias resulting from selection. Furthermore, there are some errors in cattle pedigrees due to misidentification and incorrect recording. Therefore, pedigree-based inbreeding substantially underestimates the true inbreeding rate. The substantially higher value of F_GRM_ compared to F_ROH_, is likely due to the fact that base allele frequencies were not known and F_GRM_ cannot distinguish between alleles that are IBD and IBS. The same trend was observed in the simulated data.

Correlations between the different inbreeding measurements were positive and high. The correlation between F_PED_ and F_ROH_ was in the range of previous studies [9, 12, 28] and about 0.70. Purfield et al. [9] reported a correlation of about 0.75 between ROH and pedigree-based inbreeding coefficients for cattle using medium and high density SNP panels. A correlation of about 0.70 has been reported between ROH- and pedigree-based inbreeding coefficients for Italian Holstein cattle using a minimum number of 15 SNP (approximately 1 Mb) [28]. The differences between studies can be attributed to differences in population structure, pedigree depth, pedigree completeness and strategies to define ROH. With increasing pedigree depth, the differences between F_PED_ and F_ROH_ are expected to decrease. ROH captures both ancient and recent relationships by observing shared chromosomal segments, while pedigree-based relationships reflect recent inbreeding, which is only an expectation based on the recorded pedigree. The high correlation of F_PED_ and F_ROH_ indicates that most of the inbreeding is recent and can also be attributed to the relatively complete pedigree.

The correlation between F_GRM_ and F_PED_ using an allele frequency of 0.5 was 0.64. VanRaden et al. [23] obtained a correlation of 0.59 when an allele frequency of 0.5 was used. Hayes and Goddard [29] reported a correlation of 0.69 using 0.5 frequencies for Australian Angus bulls. Similar to previous results [12, 28], the estimates of average F_GRM_ was higher than those of F_ROH_ and F_PED_. This was also in agreement with the simulation study, in when base allele frequencies deviated from 0.5. This may be because in the genomic relationship based inbreeding method alleles that are IBD and IBS cannot be distinguished in F_GRM_. In the simulation, when true base allele frequencies were used, F_GRM_ values were in the same scale as true inbreeding coefficients. In agreement with current results, Toro et al. [30] also suggested using the true allele frequency to express genomic coefficients on the same scale as pedigree-based coefficients. However, simulation results showed high correlation between F_GRM-Fixed_ and F_TRUE_ even in populations with base allele frequencies different from 0.5. Similar to our simulation results, the correlation between F_ROH_ and F_GRM_ was extremely high (*r* = 0.94) in the Holstein population. Bjelland et al. [22] reported a correlation of 0.81 between F_ROH_ and F_GRM-Fixed_. Using a high density panel, Zavarez et al. [31] showed that the correlation between F_GRM_ and F_ROH_ decreased by increasing the ROH length, probably due to the property of the genomic relationship matrix, which is based on individual loci averaged across the genome, whereas F_ROH_ is based on chromosomal segments. Zavarez et al. [31] reported correlations of 0.74 and 0.41 for ROH greater than 0.5 Mb and 16 Mb, respectively. The genomic relationship matrix is expected to be a better indicator of relatedness between individuals [7] and, therefore, the high correlation obtained between F_ROH_ and F_GRM_ in simulation and real data would support the expectation that F_ROH_ will provide an accurate measure of relatedness, and is a better indicator for the true level of inbreeding.

While ROH based methods have been used in this study to more accurately measure and characterize inbreeding, they could have other important uses in future. Genomic selection can increase the number of ROH that have deleterious effects as well as increase ROH that have positive effects on selected traits because they contain favorable alleles. It would therefore be useful to characterize the effect on selected traits, either positive or negative, of ROH segments that are relatively common in the population, and use the resulting information for more effective selection and mating, and for the discovery of causative mutations.

Here, estimates of F_ROH_ and F_GRM_ were obtained using 50 k chip data. Although it might be expected that using higher marker density would lead to an increase in the accuracy of inbreeding estimations, there is some evidence that this is not the case, at least when the effective population size is low [32]. In fact, differences in correlations between F_ROH_ and F_PED_ when using the 50 k chip versus using high density cattle panel have been found to be small [11]. Interestingly, Zhang et al. [13] observed that detecting ROH based on a 50 k chip provides estimates of homozygosity similar to ROH from sequence data. Those authors concluded that in the absence of full sequence data, ROH based on 50 k can be used to access homozygosity levels in individuals. Marras et al. [28] showed that in populations with high linkage disequilibrium and recent inbreeding, a medium density panel may be sufficient for estimating inbreeding coefficients.

##### Runs of homozygosity

The average length of the genome within ROH using a minimum window size of 20 SNP (or 1 Mb) was about 299.6 Mb per animal. The average ROH length in animals born in 2016 was about 4.54 Mb. This is much higher than values reported in Italian Brown Swiss and Holstein populations (3.9 and 3.6 Mb, respectively) [28]. This may reflect higher recent inbreeding in North American Holstein population compared to Italian populations.

The average total number of ROH (82.3 ± 9.83) per animal was in close agreement with that in Italian Holstein cattle (81.7 ± 9.7) [28]. The relative frequencies of ROH in different length classes were about 43.5% (shorter than 2 Mb), 23.9% (2–4 Mb), 17.7% (4–8 Mb), 10.5% (8–16 Mb) and 4.7% (longer than 16 Mb). In agreement with previous studies [12, 28], short ROH segments (shorter than 2 Mb) were found more frequently than longer ROH (Fig. 5). Corresponding values in Italian Holstein were about 56.9%, 20.8%, 11.9%, 7.2% and 3.7%, respectively [28]. Generally, the length of ROH has an exponential distribution [24]. Short ROH results from homozygosity of ancient haplotypes and reflects the ancient relationship, while medium and long ROH mostly arise from relatively recent relatedness within populations. Different frequencies observed in two populations may be because of different population structures, history of populations, and selection strategies. Although short ROH were more frequent than longer ROH, they had small contribution to the overall autozygosity in the genome. Short ROH (shorter than 2 Mb) covered only a small proportion of the total genome length (approximately 12%).

**Fig. 3:**
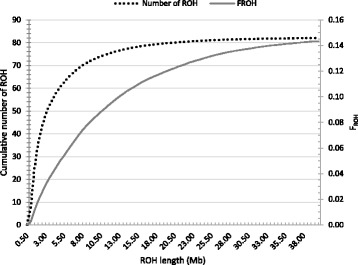
Cumulative number and inbreeding based on runs of homozygosity (F_ROH_) in animals born in 2016 (dotted and solid lines represent number of ROH and inbreeding across the ROH length, respectively), using SNP1101 software (minimum window size = 20SNP, genotype error = 0.001)

Figure 3 shows the cumulative number of ROH and F_ROH_ in different ROH lengths in the year 2016. ROH segments less than 6 Mb accounted for approximately 78% of all segments and contributed about 0.06 of the total inbreeding (Fig. 3). When excluding ROHs less than 6 Mb, average F_ROH_ became very close to average F_PED_. Based on these results, it seems that a large proportion of inbreeding in North American Holstein is the result of a reduced effective population size due to recent selective breeding programs, in which only a few top bulls contribute the most to the population gene pool.

##### Effect of the selection program

**Fig. 4:**
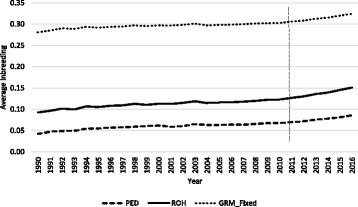
Average estimates of inbreeding per year in North American Holstein cattle. Inbreeding based on pedigree (PED), inbreeding derived from runs of homozygosity (ROH), inbreeding estimated from the genomic relationship matrix using an allele frequency of 0.5 (GRM_ Fixed). ROH was estimated using SNP1101 with minimum window size = 20SNP, genotype error = 0.001. Gray dashed line represent the start of genomic selection

With the implementation of genomic evaluations in North America in 2009, progeny test schemes aimed at identifying elite sires have changed. Genomic selection has enabled the selection of genetically superior animals at an early age with satisfactory accuracy. Even though the accuracy of estimated genomic breeding values of young animals is not as high as those from progeny tested bulls, the advantage of genomic selection due to higher selection intensity and reduction in generation interval is well documented [3]. Nowadays, genomic young bulls are being widely used worldwide and the use of progeny tested bulls has reduced drastically, resulting in a faster generation turnover. The generation interval has decreased from 6 to 3, and 4 to 3 for sire and dams since 2009 [33], respectively. Fig. 4 shows the trend in the average inbreeding of genotyped animals by year of birth. In 2011, when the first progenies of genomic sires were born, the mean ROH-based inbreeding coefficient was estimated at 0.12. Then mean inbreeding coefficient increased until 2016, when it reached 0.15.

For animals born prior to 2010, before initial use of genomic young sires in North America, the average F_ROH_ increased about 0.001 per year, while after implementation genomic selection average F_ROH_ increased about 0.005 per year. The average annual increase of pedigree-based inbreeding after implementation of genomic selection was also higher (0.003 vs. 0.001). These results show that average inbreeding is increasing at a faster pace than before implementation of genomic selection in North American Holsteins. Therefore, managing inbreeding has become even more important.

**Fig. 5:**
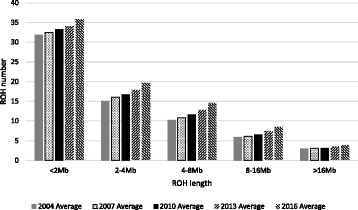
Average number of runs of homozygosity (ROH) in different ROH categories for different birth years (from 2004 to 2016), using SNP1101 software (minimum window size = 20SNP, genotype error = 0.001). The SE of the means ranged from 0.05 to 0.13

Genomic selection has resulted in a higher number of ROH segments, regardless ROH size, compared to traditional selection. The rate of increase in the number of ROH post genomic selection was about 2.1 ± 0.05 per year while this was only 0.57 ± 0.01 prior to genomic selection. In agreement with simulation results, short and medium ROHs (shorter than 16 Mb) became more frequent after implementing genomic selection than before genomic selection (Fig. 5 and Additional file 1: Table S1). While long ROH were not reduced after genomic selection, the number of animals with at least one long ROH has decreased significantly per year (7.32 ± 2.13 vs. 89.31 ± 6.94). The use of genomic selection has resulted in increasing the number of ancestors contributing to the next generations, and therefore has reduced the close relationships in the population.

## Conclusions

Simulation results confirmed that pedigree-based inbreeding may underestimate true autozygosity in selected populations. The amount of the bias depends on the accuracy and intensity of selection. ROH had the highest relationship with true inbreeding in all simulated populations. A minimum ROH length of 20 to 50 SNP using SNP1101 gave the most accurate and efficient estimates of inbreeding in all populations. In the North American Holstein population, the number of ROH has increased noticeably over the last few years as a consequence of selection, especially after implementation of genomic selection. The distribution of ROH was in agreement with previous studies, shorter ROH were found more frequently than medium and long ROHs. As a result of genomic selection, ROH frequency and distribution has changed and the rate of increase in inbreeding per year has become steeper. Therefore, the management of inbreeding has become a more urgent issue than in the past.

## Methods

### Simulation

#### Population structure

A base population consisted of 500 females and 50 males was simulated using QMSim [34]. Sixty overlapping generations were generated by mating 50 sires at random to 500 dams. Each dam produced two progeny in each generation. Sire and dam replacement rates were 0.5 and 0.3, respectively in each generation. Offspring were chosen as parents for the next generation using four different types of selection (random, phenotypic, BLUP-EBV and GEBV selection).

The BLUP-EBV were estimated using Henderson’s mixed linear equations implemented in QMSim for an individual animal model, considering the true simulated additive genetic variance. A single trait with heritability of 0.3 and phenotypic variance of 1.0 was simulated. The true breeding value of an animal was calculated as the sum of the QTL additive effects. The phenotypes were generated by adding random residuals to the true breeding values. The whole simulation process was repeated 20 times.

#### Genome

In order to measure true inbreeding, a gene dropping simulation was carried out [35]. The gene dropping approach is a simple method based on the idea of gene flow through a pedigree. For this purpose, first, the genome including 29 autosomal chromosomes with a total length of 2496 cM [36] was simulated in QMSim. In total 54,000 bi-allelic SNP loci and 750 multi-allelic QTL loci were randomly placed across the genome. QTL effects were assumed to be additive. The additive QTL effects were sampled from a gamma distribution with a shape parameter of 0.4, based on results from Hayes & Goddard [37]. As sampling from the gamma distribution results in positive effects, the sign of the QTL effect was sampled to be positive or negative with equal probability. The mutation rate was assumed to be zero.

**Fig. 6:**
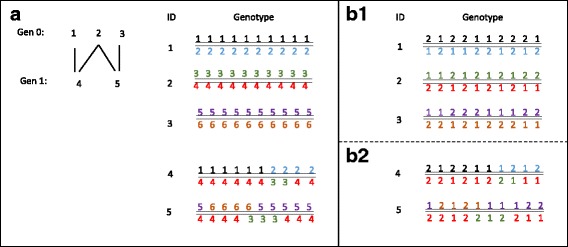
Description of the gene dropping approach for 5 animals with 10 SNPs. **a**) Six unique allele numbers (2* number of individuals in generation zero) were assigned to the 3 founder animals in generation zero. The alleles then dropped through pedigree following Mendelian inheritance and considering an average of 1 crossover per 100 centiMorgans **b1**) Unique alleles for each locus were randomly recoded to one or two in the generation zero using a random number generator, aiming to the desired allele frequency in base population. **b2**) Alleles of descendants were assigned based on inheritance in the recorded pedigree in a)

In order to trace alleles from founders and to measure true inbreeding, starting alleles in generation zero were defined to be unique at each locus of the base population. So a number of 1100 unique alleles were randomly assigned to the 550 founder animals in generation zero and dropped at random to their progenies following Mendelian inheritance. In a second step, to mimic bi-allelic SNP, unique alleles for each locus were randomly recoded to one or two in the base population using a random number generator. To evaluate the effect of base allele frequency on estimation of genotype relationship-based inbreeding, two populations with different distributions of base minor allele frequencies were created; 1) a population with uniformly distributed base allele frequencies (ranging from 0 to 0.5) [Additional file 1: Figure S1] and, Fig. 2) a population with equal base allele frequencies (approximately 0.5) [Additional file 1: Figure S2]. The assignment of alleles of all descendants, based on the actual pedigree, were done following Mendelian segregation rules. Schematic representation of the gene dropping approach is illustrated in Fig. 6.

### Runs of homozygosity detection and distribution

The ROH segments were identified using SNP1101 [38], PLINK [39] and BCFtools [25] software. In total, three alternatives were tested using PLINK; 1 heterozygote option (one heterozygote allowed per homozygosity window) × 3 different sliding window sizes in number of SNPs: 20, 35, 50. The threshold to call a ROH was the same as for the sliding window. Minimum window size of 5, 20, 35, 50 SNPs were also tested in SNP1101. The minimum SNP density in SNP1101 was set to 1 SNP every 50 kb to ensure low SNP density did not affect ROH length and, therefore, minimum ROH lengths of 0.25, 1, 1.75, and 2.5 Mb were set for window sizes 5, 20, 35 and 50 SNP, respectively. Considering the effect of genotyping errors on ROH detection [11], the genotyping error rate of 0.1% was chosen based on expected error rate in genotypes. The criteria for defining genomic regions as ROH using SNP1101 were chosen to facilitate comparison of results with those obtained with PLINK. BCFtools uses HMM approach that allows the inclusion of SNP positions from the genetic map, which are used to determine the transition probabilities based on likely recombination events between two SNPs.

For each animal in each population, the total number of ROH detected and the average, maximum, and minimum length of ROH (in SNP) were calculated. The distribution of length of ROH within populations was assessed using box plots.

### Inbreeding coefficient estimation

The pedigree-based inbreeding coefficients were estimated using the complete pedigree information in QMSim. True inbreeding (F_TRUE_) was defined as the true proportion of autozygous loci in an individual’s genome. F_ROH_ inbreeding coefficients were calculated as the sum of ROH lengths of an individual divided by the total length of the autosomes (in number of SNP). In addition*,* inbreeding coefficient (F_GRM_) from the diagonal of the genomic relationship matrix (GRM) was obtained using the method described by VanRaden et al. [7] using SNP1101. The GRM matrix was calculated as:

$$ \mathbf{G}=\raisebox{1ex}{$\mathbf{Z}{\mathbf{Z}}^{\prime }$}\!\left/ \!\raisebox{-1ex}{$2\sum p\left(1-p\right)$}\right. $$

where **Z** is a matrix containing values 0 − 2p for homozygotes, 1 − 2p for heterozygotes, and 2 − 2p for opposite homozygotes, where p is the allele frequency of SNP i. As F_GRM_ depends on allele frequencies, it is important to examine how varying allele frequencies affect estimated inbreeding coefficients. To investigate the impact of using different allele frequencies on F_GRM_, we used: (1) known base allele frequency, and (2) equal allele frequency of 0.5.

To compare different inbreeding coefficient estimates, the mean and standard errors of means over 20 replicates were calculated. In addition, correlation analyses between different inbreeding coefficients were performed to assess the strength of the association between the different estimates.

### Real data

The dataset used to investigate ROH and inbreeding coefficients consisted of 41,585 North American Holstein cattle including 21,156 bulls and 20,431 cows born between 1990 and 2016. Of these, 28,004 animals had 50 k genotypes, 5537 animals had genotype density between 10 k and 50 k and the rest were genotyped with density between 7 k and 10 k. Un-genotyped loci for animals with a lower density panel were imputed to 50 k denser SNP panel using FImpute [27]. The dataset included all genotyped animals available before 2003 and random samples of 2000 animals with a pedigree completeness index larger than 0.90 (going 8 generations back) selected in each year after 2003. A total of 44,369 SNPs used for official genomic evaluation in Canada were used in the current study. A detailed description of the genotype quality control was given by Wiggans et al. [40]. The kinship command in SNP1101 was used to calculate both the genomic and the pedigree-based inbreeding. For calculation of genomic-based inbreeding, an allele frequency of 0.5 was used as suggested by VanRaden et al. [23]. F_ROH_ was calculated using the best performing method based on simulation results.

## Additional file

Additional file 1: Table S1. Runs of homozygosity (ROH) statistics in North American Holstein cattle for different birth years, using SNP1101 software (min window size = 20SNP, genotype error = 0.0001). **Figure S1.** Distribution of minor allele frequency in the base generation of simulated populations (uniform allele frequencies). **Figure S2.** Distribution of minor allele frequencies in the base generation of simulated populations with equal allele frequency (*p* = 0.5). (DOCX 302 kb)

## Abbreviations

A: Additive relationship matrix
BLUP: Best linear unbiased prediction
BLUP-EBV: BLUP estimated breeding values
F_GRM_: Inbreeding values based on genomic relationship
F_GRM-Base_: Inbreeding values based on genomic relationship with true allele frequency obtained from the base population
F_GRM-Fixed_: Inbreeding values based on genomic relationship with allele frequency of 0.5
F_PED_: Inbreeding values based on pedigree
F_ROH_: Inbreeding values based on runs of homozygosity
F_TRUE_: True inbreeding
GEBV: Genomic breeding values
GRM: Genomic relationship matrix
HMM: Hidden Markov Model
IBD: Identical by descent
IBS: Identical by state
QTL: Quantitative trait locus
ROH: Runs of homozygosity
SNP: Single nucleotide polymorphism
