# Supplementary material for: Inbreeding and runs of homozygosity before and after genomic selection in North American Holstein cattle
Source: BMC Genomics. 2018 Jan 27;19:98. doi: 10.1186/s12864-018-4453-z (PMC5787230; doi:10.1186/s12864-018-4453-z)
Supplement: Additional file 1: Table S1. — Runs of homozygosity (ROH) statistics in North American Holstein cattle for different birth years, using SNP1101 software (min window size = 20SNP, genotype error = 0.0001). Figure S1. Distribution of minor allele frequency in the base generation of simulated populations (uniform allele frequencies). Figure S2. Distribution of minor allele frequencies in the base generation of simulated populations with equal allele frequency (p = 0.5). (DOCX 302 kb) [file 12864_2018_4453_MOESM1_ESM.docx]

**Table S1**. Runs of homozygosity (ROH) statistics in North American Holstein cattle for different birth years, using SNP1101 software (min window size = 20SNP, genotype error=0.0001).

| Year | Number of ROH ± standard deviation | ROH length (Mb) | | | | |  |
| --- | --- | --- | --- | --- | --- | --- | --- |
|  |  | **Less than 2 ±**  **standard deviation** | **2 to 4**  **± standard deviation** | **4 to 8**  **± standard deviation** | **8 to 16**  **± standard deviation** | **Greater than 16 ± standard deviation** | |
| 1990 | 56.99 ± 7.95 | 29.85 ± 5.47 | 13.03 ± 3.66 | 7.42 ± 3.07 | 4.30 ± 2.30 | 2.39 ± 2.06 |  |
| 1991 | 58.60 ± 8.13 | 30.34 ± 5.34 | 13.32 ± 3.57 | 7.78 ± 2.99 | 4.49 ± 2.45 | 2.66 ± 2.21 |  |
| 1992 | 60.46 ± 8.71 | 31.30 ± 5.58 | 13.51 ± 3.93 | 8.10 ± 3.16 | 4.85 ± 2.53 | 2.70 ± 2.20 |  |
| 1993 | 60.24 ± 8.63 | 31.13 ± 5.65 | 13.77 ± 3.88 | 8.12 ± 3.17 | 4.62 ± 2.44 | 2.60 ± 2.12 |  |
| 1994 | 62.09 ± 9.38 | 31.25 ± 5.63 | 14.18 ± 3.98 | 8.60 ± 3.28 | 5.06 ± 2.52 | 3.00 ± 2.29 |  |
| 1995 | 62.02 ± 9.15 | 31.15 ± 5.88 | 14.07 ± 3.92 | 8.93 ± 3.26 | 5.01 ± 2.51 | 2.87 ± 2.16 |  |
| 1996 | 62.82 ± 8.97 | 31.32 ± 5.86 | 14.14 ± 4.01 | 9.08 ± 3.35 | 5.35 ± 2.65 | 2.93 ± 2.15 |  |
| 1997 | 63.53 ± 8.40 | 31.12 ± 5.75 | 14.73 ± 4.03 | 9.36 ± 3.26 | 5.32 ± 2.52 | 2.99 ± 2.05 |  |
| 1998 | 64.12 ± 8.70 | 31.32 ± 5.84 | 14.43 ± 3.85 | 9.70 ± 3.35 | 5.51 ± 2.49 | 3.17 ± 2.10 |  |
| 1999 | 63.98 ± 8.52 | 31.52 ± 5.81 | 14.50 ± 3.98 | 9.38 ± 3.24 | 5.60 ± 2.51 | 2.97 ± 2.03 |  |
| 2000 | 64.88 ± 8.54 | 31.59 ± 5.65 | 14.94 ± 4.11 | 9.70 ± 3.26 | 5.53 ± 2.53 | 3.11 ± 2.06 |  |
| 2001 | 65.50 ± 8.72 | 32.05 ± 5.70 | 14.77 ± 4.12 | 10.00 ± 3.34 | 5.72 ± 2.64 | 2.96 ± 2.04 |  |
| 2002 | 66.10 ± 8.62 | 31.87 ± 5.85 | 15.19 ± 4.14 | 10.10± 3.31 | 5.88 ± 2.58 | 3.05 ± 2.03 |  |
| 2003 | 66.86 ± 8.31 | 31.82 ± 6.87 | 15.42 ± 4.12 | 10.27± 3.34 | 6.10 ± 2.64 | 3.25 ± 2.12 |  |
| 2004 | 66.12 ± 8.25 | 31.85 ± 5.67 | 15.02 ± 4.08 | 10.27± 3.23 | 5.94 ± 2.60 | 3.05 ± 1.97 |  |
| 2005 | 66.61 ± 8.33 | 31.54 ± 5.73 | 15.50 ± 4.22 | 10.48± 3.33 | 6.00 ± 2.60 | 3.08 ± 1.98 |  |
| 2006 | 67.22 ± 8.08 | 31.89 ± 5.61 | 15.75 ± 4.05 | 10.54± 3.40 | 5.97 ± 2.58 | 3.09 ± 2.03 |  |
| 2007 | 68.42 ± 8.29 | 32.49 ± 5.84 | 16.01 ± 4.02 | 10.79± 3.41 | 6.06 ± 2.59 | 3.06 ± 1.99 |  |
| 2008 | 69.27 ± 8.25 | 32.58 ± 5.71 | 16.28 ± 4.14 | 11.11± 3.52 | 6.22 ± 2.57 | 3.08 ± 1.93 |  |
| 2009 | 69.82 ± 8.22 | 32.56 ± 5.65 | 16.31 ± 4.14 | 11.27± 3.42 | 6.52 ± 2.63 | 3.16 ± 2.00 |  |
| 2010 | 71.26 ± 8.41 | 33.33 ± 5.76 | 16.72 ± 4.14 | 11.60± 3.50 | 6.51 ± 2.62 | 3.10 ± 2.01 |  |
| 2011 | 71.82 ± 8.45 | 33.00 ± 5.86 | 17.06 ± 4.08 | 11.67± 3.41 | 6.82 ± 2.70 | 3.27 ± 2.03 |  |
| 2012 | 73.77 ± 8.43 | 33.77 ± 5.65 | 17.51 ± 4.23 | 12.11± 3.54 | 7.11 ± 2.78 | 3.27 ± 2.10 |  |
| 2013 | 75.59 ± 8.69 | 34.06 ± 5.95 | 17.84 ± 4.18 | 12.82± 3.73 | 7.39 ± 2.79 | 3.48 ± 2.15 |  |
| 2014 | 77.90 ± 8.93 | 34.85 ± 5.88 | 18.59 ± 4.36 | 13.33± 3.84 | 7.68 ± 3.89 | 3.46 ± 2.16 |  |
| 2015 | 80.32 ± 9.80 | 35.23 ± 6.11 | 19.24 ± 4.57 | 14.04± 3.84 | 8.12 ± 3.00 | 3.69 ± 2.24 |  |
| 2016 | 82.31 ± 9.83 | 35.80 ± 5.97 | 19.68 ± 4.77 | 14.56± 3.96 | 8.46 ± 3.02 | 3.81 ± 2.23 |  |

0.84

0.63

0.42

0.21

0.00-0.02

0.02-0.04

0.04-0.06

0.06-0.08

0.08-0.10

0.10-0.12

0.12-0.14

0.14-0.16

0.16-0.18

0.18-0.20

0.20-0.22

0.22-0.24

0.24-0.26

0.26-0.28

0.28-0.30

0.30-0.32

0.32-0.34

0.34-0.36

0.36-0.38

0.38-0.40

0.40-0.42

0.42-0.44

0.44-0.46

0.46-0.48

0.48-0.50

Frequency


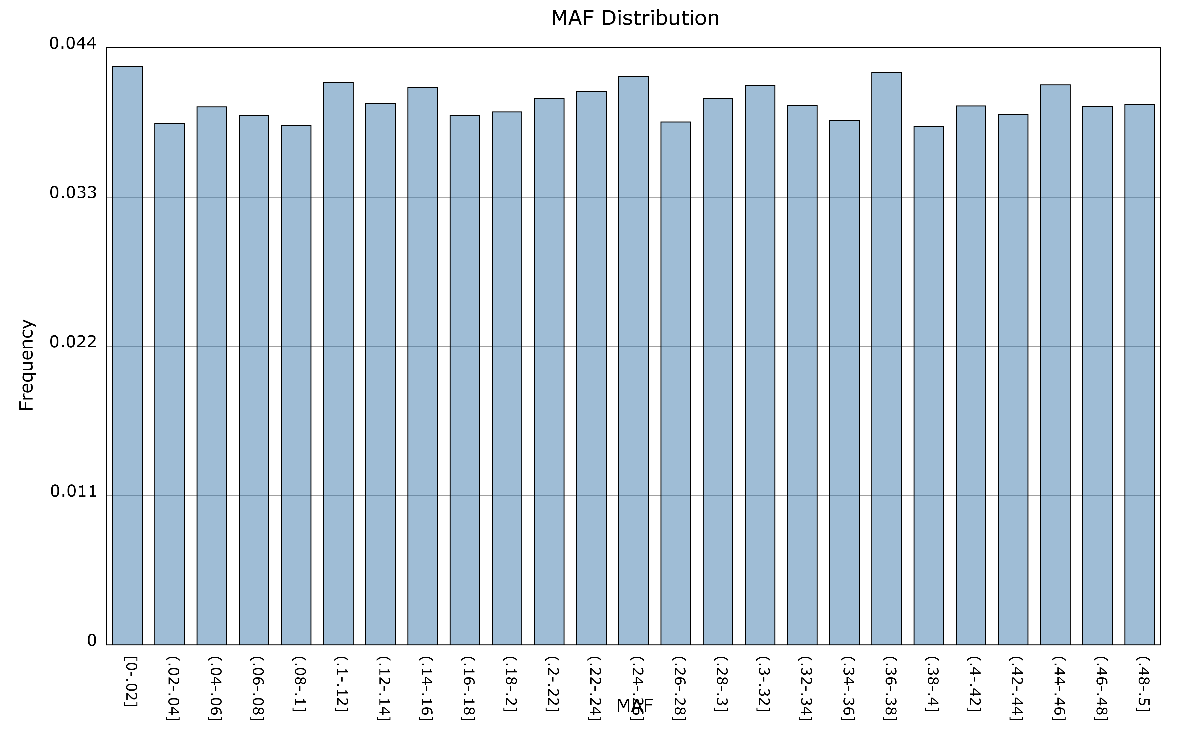


Minor allele frequency

**Figure S1**. Distribution of minor allele frequency in the base generation of simulated populations (uniform allele frequencies)

**Figure S2**. Distribution of minor allele frequencies in the base generation of simulated populations with equal allele frequency (p= 0.5)

0.84

0.63

0.42

0.21

0.00-0.02

0.02-0.04

0.04-0.06

0.06-0.08

0.08-0.10

0.10-0.12

0.12-0.14

0.14-0.16

0.16-0.18

0.18-0.20

0.20-0.22

0.22-0.24

0.24-0.26

0.26-0.28

0.28-0.30

0.30-0.32

0.32-0.34

0.34-0.36

0.36-0.38

0.38-0.40

0.40-0.42

0.42-0.44

0.44-0.46

0.46-0.48

0.48-0.50

Frequency


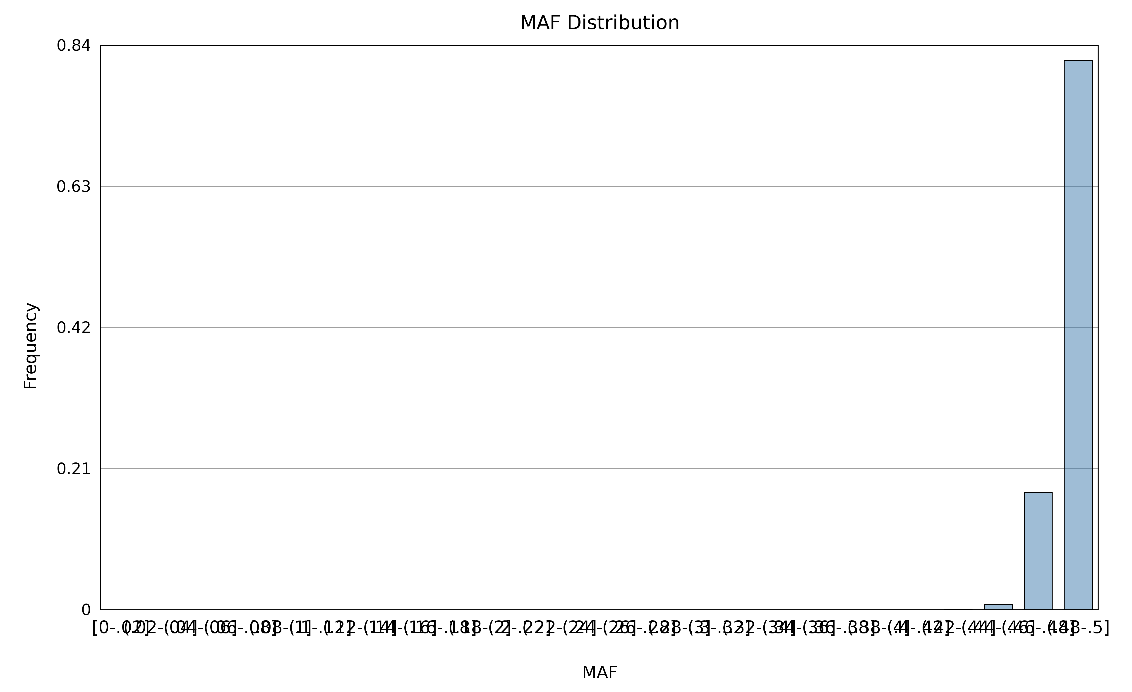


Minor allele frequency
